# Insights into the microRNA landscape of *Rhodnius prolixus*, a vector of Chagas disease

**DOI:** 10.1038/s41598-023-40353-9

**Published:** 2023-08-12

**Authors:** Paula Beatriz Santiago, Kaio Luís da Silva Bentes, Waldeyr Mendes Cordeiro da Silva, Yanna Reis Praça, Sébastien Charneau, Soraya Chaouch, Philippe Grellier, Marcos Antônio dos Santos Silva Ferraz, Izabela Marques Dourado Bastos, Jaime Martins de Santana, Carla Nunes de Araújo

**Affiliations:** 1 Pathogen-Host Interface Laboratory, Department of Cell Biology, Institute of Biology, University of Brasília, Brasília, DF Brazil; 2 Federal Institute of Goiás, Formosa, Brazil; 3 Laboratory of Protein Chemistry and Biochemistry, Department of Cell Biology, Institute of Biology, University of Brasília, Brasília, Brazil; 4 UMR 7245 Molécules de Communication et Adaptation des Micro-organismes, Muséum National d’Histoire Naturelle, CNRS, CP52, 61 rue Buffon, 0575231 Paris Cedex, France; 5 Department of Cell Biology, Scientific Illustration Center, Institute of Biology, University of Brasília, Brasília, DF Brazil; 6 Faculty of Ceilândia, University of Brasília, Brasília, DF Brazil

**Keywords:** Non-coding RNAs, Post-translational modifications, Data acquisition, Parasitic infection, Entomology

## Abstract

The growing interest in microRNAs (miRNAs) over recent years has led to their characterization in numerous organisms. However, there is currently a lack of data available on miRNAs from triatomine bugs (Reduviidae: Triatominae), which are the vectors of the protozoan parasite *Trypanosoma cruzi,* the causative agent of Chagas disease. A comprehensive understanding of the molecular biology of vectors provides new insights into insect-host interactions and insect control approaches, which are key methods to prevent disease incidence in endemic areas. In this work, we describe the miRNome profiles from gut, hemolymph, and salivary gland tissues of the *Rhodnius prolixus* triatomine. Small RNA sequencing data revealed abundant expression of miRNAs, along with tRNA- and rRNA-derived fragments. Fifty-two mature miRNAs, previously reported in Ecdysozoa, were identified, including 39 ubiquitously expressed in the three tissues. Additionally, 112, 73, and 78 novel miRNAs were predicted in the gut, hemolymph, and salivary glands, respectively. In silico prediction showed that the top eight most highly expressed miRNAs from salivary glands potentially target human blood-expressed genes, suggesting that *R. prolixus* may modulate the host’s gene expression at the bite site. This study provides the first characterization of miRNAs in a Triatominae species, shedding light on the role of these crucial regulatory molecules.

## Introduction

Insects of the Triatominae subfamily (Hemiptera: Reduviidae) are vectors of the Chagas disease agent *Trypanosoma cruzi*. While the impact of Chagas disease has decreased over the years, it continues to cause disability and death in Latin America, an endemic region where transmission primarily occurs during the blood meal of an infected insect or through the ingestion of contaminated food^1^. Moreover, due to migratory flows, the disease has been reported in non-endemic areas, where alternative routes such as blood transfusion, organ transplantation, and vertical transmission take place^1–3^. Estimates indicate a range of 6–7 million people worldwide suffer from disease-related morbidity, resulting in over 10,000 deaths annually^4–8^. *Rhodnius prolixus* is considered one of the main vectors of Chagas disease in Central America and northern South America.

The vector's bite causes vascular and tissue damage in the host, triggering a series of physiological defense mechanisms that act against pathogen invasion and blood loss, including immune response, inflammation, and hemostasis. Many studies have reported on the key effects of salivary molecules from blood-sucking insects, which are secreted during the bite to assist in the feeding process, counteracting the host's hemostasis, and modulating its immune responses^9–11^. In this context, transcriptomic and proteomic studies have unveiled the complexity of salivary content^12^. The blood ingested from the host is stored, digested, and absorbed in the gut compartments of the insect, where, upon infection, *T. cruzi* develops and replicates^13^. However, triatomine immune pathways and the details of their interactions with *T. cruzi* remain poorly understood. Insect hemolymph is a dynamic circulating tissue that mediates hormone, nutritional, and immunological homeostasis. Its composition varies depending on factors such as nutrient availability, stress conditions, and current physiological needs^14^. Hemolymph components are involved in the dynamics of the insect immunity, and in this regard, hemocytes and multiple soluble effectors work together towards a coordinated response to injury and/or control of pathogen development^15^.

Studies on gene regulation have revealed microRNAs (miRNAs) as a critical research topic. Insect miRNAs may coordinate the regulation of many protein-coding genes related to various biological processes^16^. Functional investigations have established their roles in metabolism^17–19^, development, apoptosis^20–23^, and immune responses^24,25^. MiRNAs are small non-coding RNAs (sncRNAs) with ~ 22 nucleotides in length that regulate gene expression by inhibiting translation or targeting mRNAs for cleavage at the posttranscriptional level through base pairing with the complementary untranslated regions (3′UTR) of a target mRNA sequence^16,26–28^. Conversely, miRNAs have also been reported to upregulate the expression of target genes^29–31^.

Knowledge on the roles of miRNA in arthropod hematophagous vectors is also increasing. Analyses of miRNA expression profiles in mosquitoes suggests that they are induced upon blood feeding and may regulate development and immunity^32–39^. Interactions of aae-miR-375 with *Aedes aegypti* immune-related genes have been documented and enhance Dengue virus serotype 2 infection in *Ae. aegypti* cell lines^38^. Mosquito miRNAs are also differentially expressed in the presence of the bacterial endosymbiont *Wolbachia*, where aae-miR-12 suppresses *Mct1* and *Mcm6* target genes, playing a crucial role in controlling *Wolbachia* density in mosquito cells^40,41^. Further studies have demonstrated that aae-miR-2940 downregulates the transcript levels of DNA methyltransferase (AaDnmt2), inhibiting Dengue virus replication in *Wolbachia*-infected mosquito cells^42^. Moreover, the expression of aae-miR-2940 is selectively downregulated in *Aedes albopictus* (C6/36) cells in response to the West Nile virus^43^.

Extracellular miRNAs (ex-miRNAs) have been observed in body fluids such as serum and saliva^44,45^. To protect and stabilize ex-miRNAs in the extracellular milieu, circulation occurs within vesicles or in miRNA/protein complexes^46,47^. Circulating ex-miRNAs can be delivered to target cells, mediating a direct cell–cell communication, or even a dynamic cross-species function, where the recipient cell takes up miRNAs to modulate protein production^44,48^. Regarding the vector-parasite-host interface, it was reported that a human-blood-derived miRNA, hsa-miR-150-5p, modulates mosquito immune response to facilitate flavivirus infection and transmission^49^. The specific mechanisms of ex-miRNA uptake by recipient cells, vesicles, or membrane receptors, as well as the control of this process, remain under investigation.

Although miRNAs have been described as important molecular regulators of other insect vectors, there is a lack of knowledge concerning miRNA expression in Triatominae. To address this gap, we described the miRNomes of the salivary glands (SG), gut (G), and hemolymph (H) from *R. prolixus* through sRNA-*seq*. Our analysis revealed transcriptional evidence of several known and novel miRNAs in *R. prolixus* tissues. This repertoire offers new insights into the dynamic interactions between vector/pathogens and vector/hosts and may be explored to expand our understanding of miRNA roles in triatomine biological functions.

## Results and discussion

### Overview of the sncRNAs from *Rhodnius prolixus*

To identify the miRNome from *R. prolixus* tissues, three independent libraries of small RNAs were constructed and used for high-throughput sequencing. These libraries generated over 63, 57, and 80 million raw reads from the G, H, and SG tissues, respectively. After size selection (≥ 18 nt) and filtering out of low-quality sequences and adapter contaminants, more than 61 (G), 38 (H), and 54 (SG) million processed reads were obtained (Supplementary Table S1).

**Figure 1:**
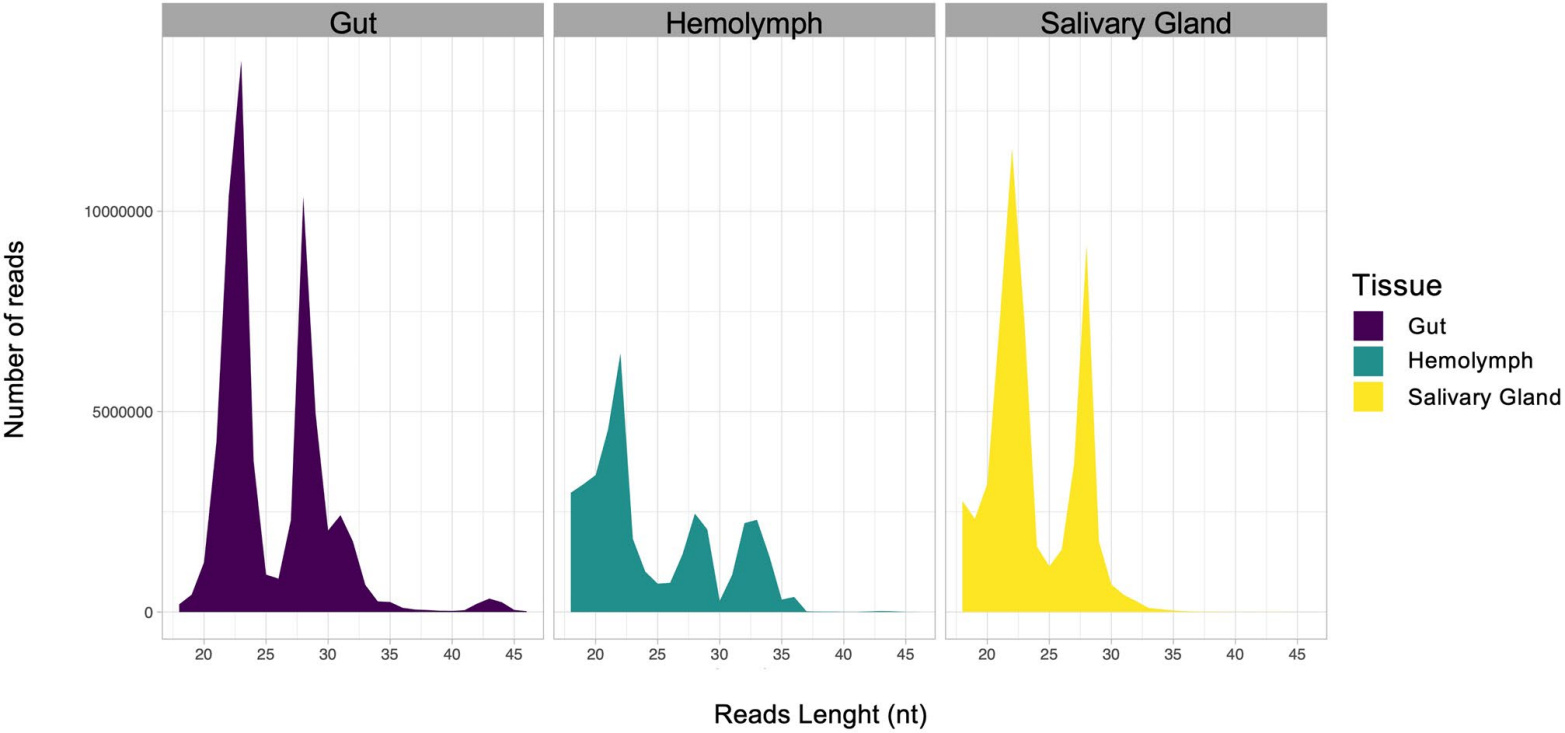
Read length distribution and abundance of small RNAs from *Rhodnius prolixus*. The graphs show the processed read numbers and size distribution (18–50 nt). The *x*-axis represents the length (nt) of the sequence. The *y*-axis represents the number of reads.

The processed reads within the three libraries (18–51 nt) were examined for read length distribution and abundance (Fig. 1). Most of the reads fell within the expected range of 20–36 nt, typical for small non-coding RNAs (sncRNAs)^50^. In the G and SG libraries, two major peaks were observed, one in the 21–23 nt range and a smaller peak in the 27–29 nt range. The length distribution of mapped reads in the H library showed smaller peaks, with the one in the 21–23 nt range being more prominent (Fig. 1). The read abundance profiles suggest that the H tissue has a relatively lower abundance of sncRNAs species. Given that the ~ 21–23 nt range corresponds to the typical size of mature miRNA, it can be concluded that miRNAs are present in a significant proportion in the three tissues analyzed. Additionally, the ~ 27 nt reads in G and SG libraries may represent piRNAs, a class of sncRNAs that typically range from 24 to 30 nt in length and can vary in abundance across different tissue types^51,52^. The two-peak feature (21–23 and 27–29 nt) has been previously reported in other insect miRNomes^53–55^.

### The miRNomes from *Rhodnius prolixus* gut, hemolymph, and salivary gland possess conserved miRNAs

**Figure 2:**
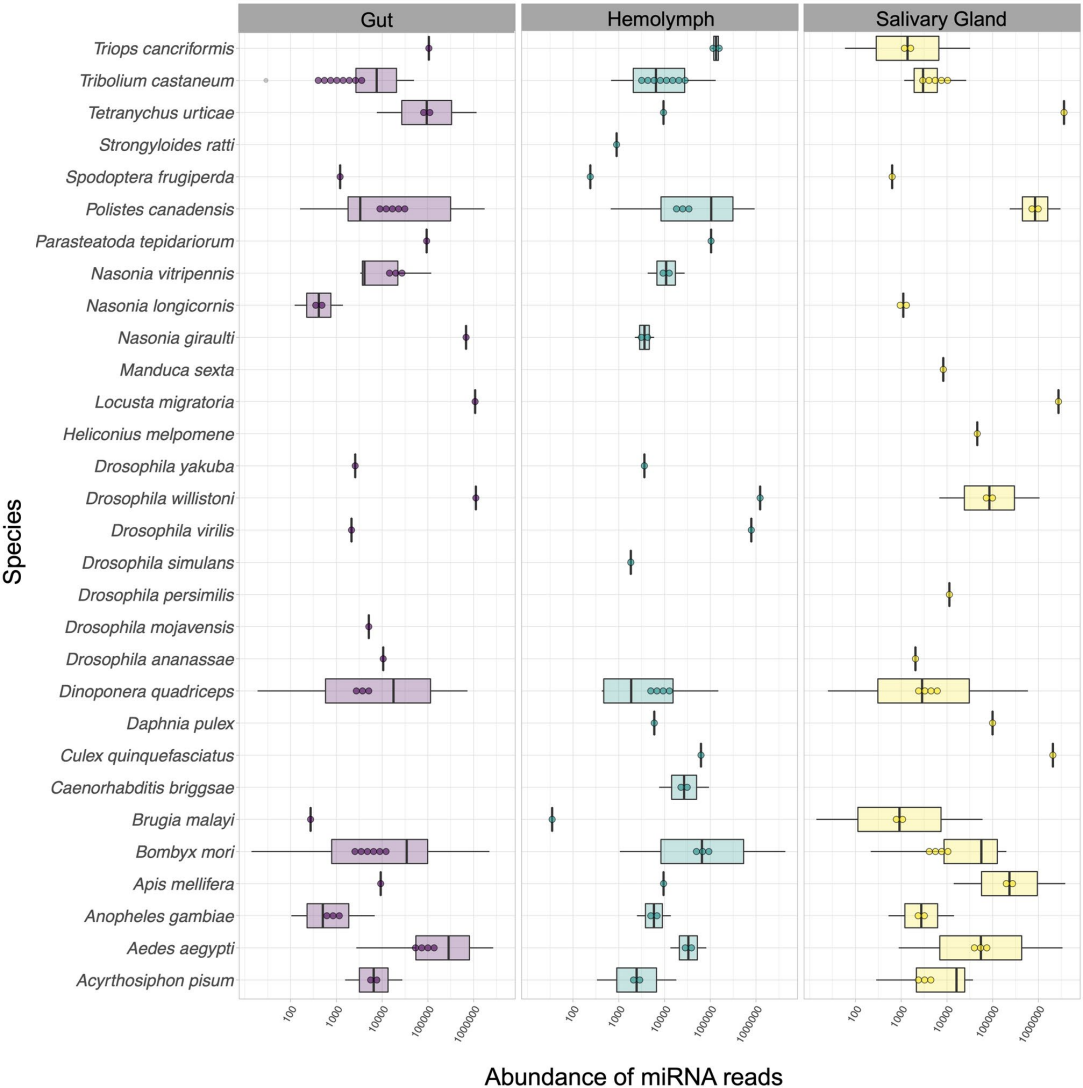
Abundance of *Rhodnius prolixus* miRNA reads aligned against known miRNAs database. The box plots illustrate hit species distribution results of *R. prolixus* genome-mapped reads aligned against a collection of known miRNAs from Ecdysozoa taxa*.* Features are based on sequence alignments and the abundance of reads from the extracted *R. prolixus* miRNAs. The box covers the interquartile interval, where 50% of the data is found. Each point represents a unique miRNA. Species are shown at the left for visualization.

**Figure 3:**
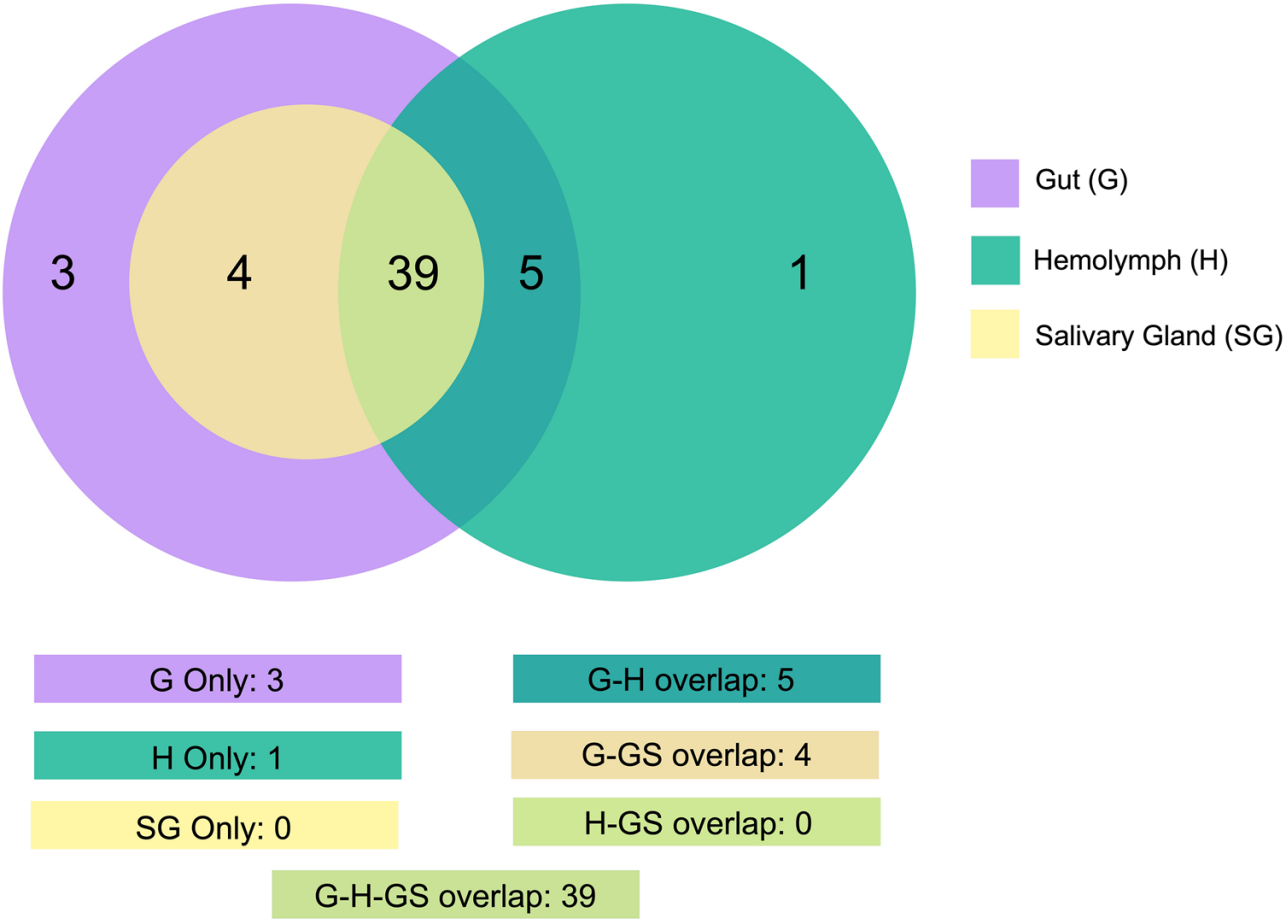
Distribution profile of *Rhodnius prolixus* conserved miRNAs. Venn diagram depicts the degree of overlap of known miRNAs from the gut, hemolymph, and salivary glands.

Genome-mapped reads were aligned against a collection of miRNA sequences from miRBase^56^ (see “Materials and methods” section); Table 1 presents the read counts and the number of the extracted *R. prolixus* miRNAs. Using the sequence similarity searching method, a total of 13,652,243 (G), 8,711,715 (H), and 21,489,863 (SG) reads were aligned, resulting in the annotation of 51 (G), 45 (H), and 43 (SG) known miRNAs. Notably, all the extracted *R. prolixus* miRNAs belonged to species within the phylum Arthropoda. The distribution of alignment hits per species and the corresponding abundance of reads from the extracted *R. prolixus* miRNAs are illustrated in Fig. 2. Overall, 52 miRNAs were identified. When comparing the resulting miRNAs extracted from the G, H, and SG tissues, a strong conservation across the tissues was observed, with an overlap of 39 miRNAs. Additionally, a small fraction of tissue-specific miRNAs was observed, with 3 miRNAs unique to G and 1 unique to H (Fig. 3). MiRBase serves as a comprehensive public repository of miRNA data, and its latest release (v.22, 2022) contains entries from 271 organisms, with a total of 48,860 mature miRNA sequences^56^. Due to the relatively limited diversity of insect sequences in miRBase, however, the recovery of conserved miRNAs in samples may have been somewhat restricted.

**Table 1:** Reads from *Rhodnius prolixus* samples identified as mature miRNAs by miRDeep2.

Sample	Total read count^1^	Mature read count^2^	Loop read count^3^	Star read count^4^	Total mature miRNAs^5^
Gut	13,652,243	13,270,733	67	381,443	51
Hemolymph	8,711,715	8,613,127	15	98,573	45
Salivary Glands	21,489,863	20,994,297	237	495,329	43

^1^The total number of genome-mapped reads that aligned against any region of a pre-miRNA as defined by miRBase.

^2^The number of genome-mapped reads that aligned against a mature sequence of a miRNA from miRBase.

^3^The number of genome-mapped reads that aligned against the loop sequence (the region between the mature and star sequence) of a miRNA from miRBase.

^4^The number of genome-mapped reads aligned against the star sequence (the sequence that aligns to the mature sequence) of a miRNA from miRBase.

^5^Number of mature miRNAs detected by miRDeep2 against miRBase (using *Ecdysozoa* taxa as reference database) within the following parameters: number of mature read count > 10; number of star read count equal to or greater than 1; miRDepp2 score equal to or exceeding the cut-off of 1; “yes” in significant randfold *p*-value.

**Figure 4:**
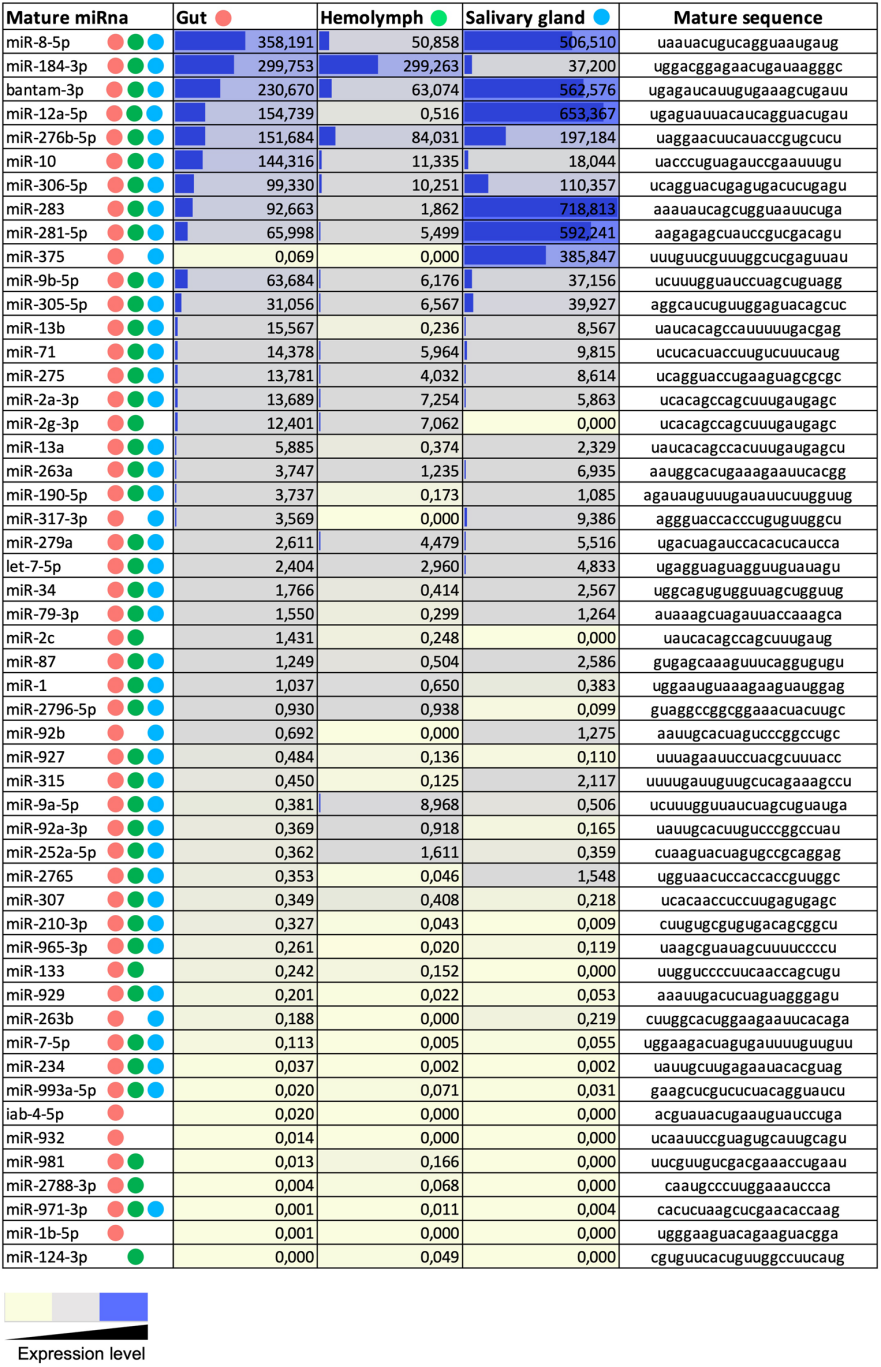
Expression levels of *Rhodnius prolixus* miRNAs. The expression levels of miRNAs are based on normalization standards and AI levels, as described in the “Materials and methods” section. The blue color represents the highly expressed conserved miRNAs, the gray color represents the mild expressed, and yellow is the less expressed, all shown with color intensities degree. Colored circles indicate the presence of miRNA in the tissue as indicated: pink, gut; green, hemolymph; blue, salivary gland. The highlighted blue bars indicate the top expressed miRNAs. The miRNA sequences are shown.

In our workflow, a single library was sequenced per tissue, which can pose challenges when discussing abundance in data analysis. To minimize this issue, we increased the number of individuals batched together and employed deep sequencing to maximize biological variability and coverage in a unique RNA-seq dataset. The expression levels of *R. prolixus* miRNAs and their nucleotide sequence can be observed in Fig. 4. A detailed analysis revealed eight up-regulated miRNAs (with an AI > 70 cut-off) in G, two in H, and eight in SG, accounting for 85%, 65%, and 94% of the mature read counts respectively, suggesting their potential crucial regulatory roles (Supplementary Fig. S1). These abundant miRNAs (miR-8-5p; miR-184-3p; bantam-3p; miR-12a-5p; miR-276b-5p; miR-10-5p; miR-306-5p; miR-283; miR-281-5p) are shared among the three tissues, although exhibiting different expression patterns (Fig. 4). Specifically, miR-276b-5p is up-regulated across all three tissues, while miR-8-5p and bantam were up-regulated in G and SG, and miR-184-3p in G and H. It has been previously suggested that insects possess a conserved miRNA repertoire comprising 65 families with minimal variation^57^. Most miRNAs identified in this study belong to this insect-conserved collection, which includes the ubiquitously up-regulated miR-8-5p, bantam, and miR276b-5p. Deep-sequencing studies have consistently demonstrated a strong conservation of miRNAs across different insect species^57^.

Interestingly, the sum of AI values for these enriched miRNAs (G: AI = 1531; H: AI = 383; SG: AI = 3726) revealed that the highest expression levels are observed in SG, surpassing those in G by more than double and H by sevenfold. A relevant aspect to note is that miRNAs from cellular fractions and ex-miRNAs were extracted together as a single sample, making it challenging to determine the contribution of each miRNA to the dynamic miRNome profiles.

Determining the physiological concentration of miRNAs and elucidating the key players involved in changes in miRNAs levels depends on physiological state and environmental responses^58,59^. The expression pattern of a specific tissue miRNome delineates the mRNA collection under its influence, shedding light on the biological pathways that may be regulated. Over millions of years, triatomines have evolved blood-feeding behavior to ensure a successful acquisition of the blood meal, suggesting that miRNAs from the SGs may also play specific roles, contributing to gene regulation at a cross-species level within vector-host and even vector-pathogen-host interactions. Indeed, ex-miRNAs have been reported in mosquitoes acting in this interface, potentially contributing to the manipulation of vertebrate host, and influencing the transmission of pathogens^60,61^.

*Drosophila* has been used as a model organism to decipher miRNA functions due to the conservation of its genes and regulatory pathways throughout evolution. Some biological functions were proposed regarding the four ubiquitously up-regulated miRNAs in *R. prolixus* tissues (miR-8, bantam, miR-184 and miR-276). miR-8 is highly conserved and plays a variety of roles related to growth and development, regulating multiple peptide hormones of body development^62,63^. It was reported that miR-8 modulates neuronal development by regulating the morphology of presynaptic arbors at the *Drosophila* neuromuscular junction through a postsynaptic mechanism^64^. In addition, atrophin is a tuning target of miR-8. Silencing of miR-8 causes neurodegeneration and consequently development defects^65^. Moreover, miR-8 and miR-2 are critical for ecdysone-induced chitin biosynthesis in the hemipteran insect *Nilaparvata lugens*^66^. Here, *R. prolixus* miR-8 is up-regulated, a trait already demonstrated in the miRNomes of other hematophagous insects like *Anopheles gambiae* and *An. funestus* mosquitoes^36,37^.

Conserved bantam also controls cell and tissue growth, mostly regulating the production of ecdysone, a hormone tightly controlled during larval development. This miRNA mediates the insulin-dependent regulation of ecdysone, acting as a buffering mechanism to adjust growth in response to nutrient availability^67^. Bantam might also be a positive hematopoietic regulator in the stem cell-like niche, by functioning upstream of the insulin signaling pathway^68^. Furthermore, it may prevent apoptosis by downregulating the apoptotic gene *hid* or even targeting the *clk* gene, regulating circadian rhythms in *Drosophila*^69,70^. MiR-184 regulates several distinct steps during oogenesis and early embryogenesis of *Drosophila* over multiple biological processes, targeting components involved in the regulation of development^71^.

A role in neural circuits of *Drosophila* underlying naive olfactory responses and conditioned odor memory was proposed for miR-276a. It was demonstrated that DopR, a type-one dopamine receptor, is a functional downstream effector of miR-276a^72^. Interestingly, miRNA expression profiles of infected and uninfected midguts from *Aedes albopictus*, an important vector for dengue virus, revealed miR-276 is up-regulated in infected mosquitoes and enhanced dengue virus replication in C6/36 cells^73^, a result that reinforces the possible unusual roles for arthropod vector miRNAs.

### Validation of miRNA expression by qPCR

The enrichment of selected miRNAs was analyzed by RT-qPCR amplification. A subset of five highly- and two lowly-abundant miRNAs from the SGs was selected for correlation analysis using slRT-PCR approach^74^. The Ct values obtained for miR-283, miR-12a, miR-281, bantam, and miR-8 (up-regulated) were much lower than those for miR-315 and miR-92b (down-regulated). The correlation between miRNA abundance (read counts) and Ct values was measured and is presented in Supplementary Fig. S2. The obtained data show RT-qPCR Ct values that reflect the read abundance in small RNA-seq analysis.

### The novel miRNAs from *Rhodnius prolixus* gut, hemolymph, and salivary glands

**Figure 5:**
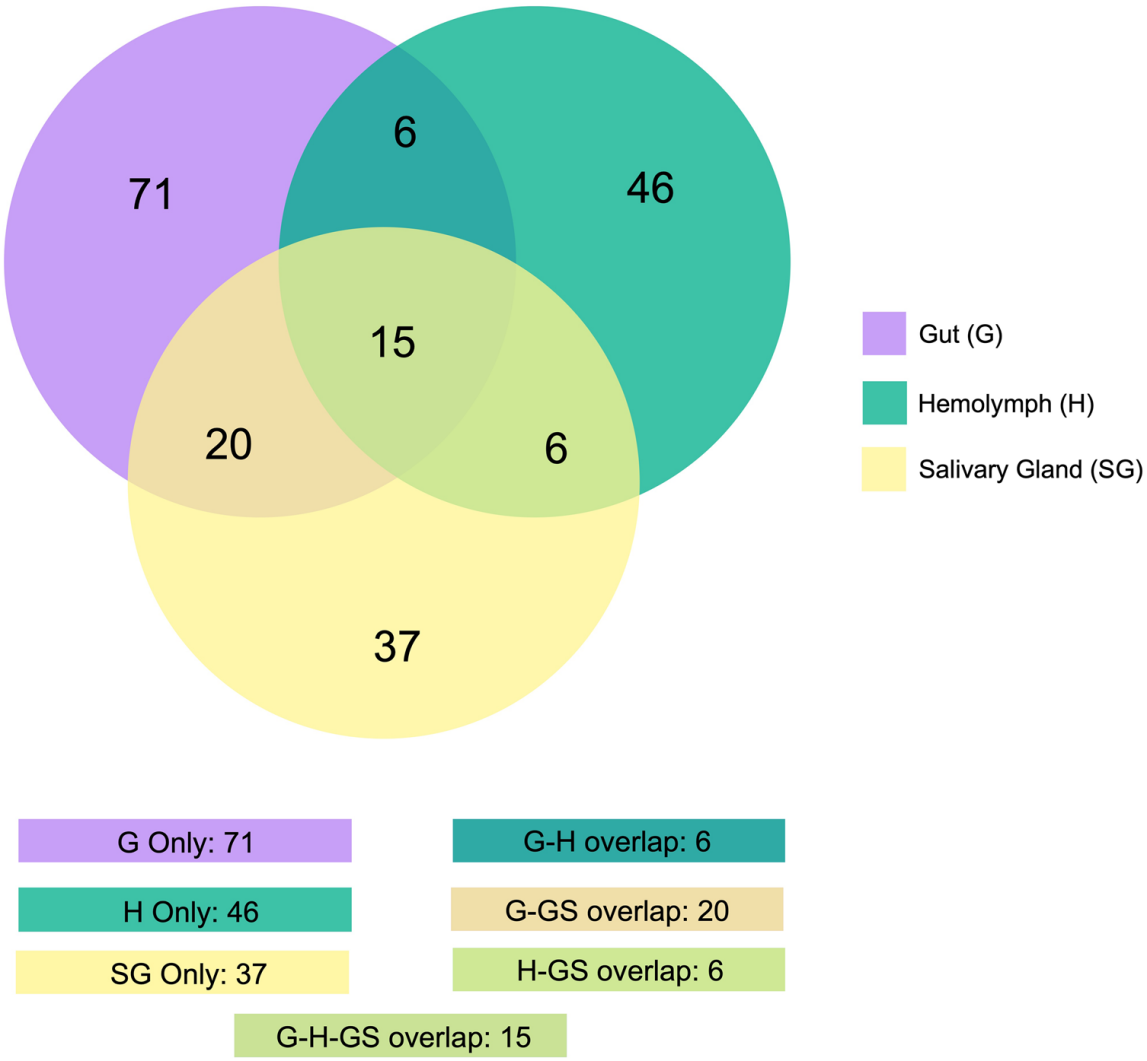
Distribution profile of *Rhodnius prolixus* novel miRNAs. The Venn diagram depicts the degree of overlap of novel mature miRNAs from the gut, hemolymph, and salivary glands.

Prediction of novel miRNAs is based on secondary structures and lack of homology with miRNAs in other species^75^. Two hundred and one candidates were predicted as novel miRNAs in *R. prolixus* miRNomes. The subsets of novel miRNAs in G, H, and SG tissues consist of 112, 73, and 78 unannotated members, respectively. Supplementary Fig. S3 presents the predicted secondary structure of the two most abundant *R. prolixus* novel miRNAs precursors in each tissue. A comparative analysis among G, H, and SG tissues showed an overlap of 15 miRNAs. Furthermore, 71 were specific to G, 45 to H, and 37 to SG. In this regard, miRNA genes evolve easily, and targets may be also easily gained and lost^76^. Most of these novel non-conserved miRNAs were found to be expressed at low levels, a trend that may be related to more specific biological roles^76^. The read counts and the total number of the predicted novel miRNAs from *R. prolixus* samples can be seen in Supplementary Table S2. The overlap analysis of these three subsets can be observed in Fig. 5.

### The sncRNA collection from *Rhodnius prolixus* is enriched with miRNA reads and possesses tRNA- and rRNA-derived RNA fragments

By aligning the genome-mapped reads against a collection of sncRNAs from Triatominae, we successfully classified the extracted sncRNA types, which included snoRNA, snRNA, tRNA- and rRNA-derived fragments. The *R. prolixus* miRNA sequences (both conserved and novel) obtained from our analysis were also included in this dataset to estimate the diversity and abundance distribution of sncRNA types within the three libraries. Supplementary Fig. S4 illustrates the diversity distribution of the identified sncRNA types from *R. prolixus* as a percentage-based representation. Tissues are mainly composed of 52% (G), 51% (H), and 54% (SG) rRNA, followed by 31% (G), 35% (H), and 20% (SG) tRNAs. With the advent of high-throughput sequencing, several sncRNA types, including tRNA- and rRNA-derived RNA fragments, have been identified^77,78^. For instance, rRNA-derived RNA fragments were reported in the small RNA sequencing library of the *Amblyomma testudinarium* tick^79^. The diversity of sncRNA types found here reflects these ongoing findings, suggesting these fragments are not random intermediates or degradation products.

**Figure 6:**
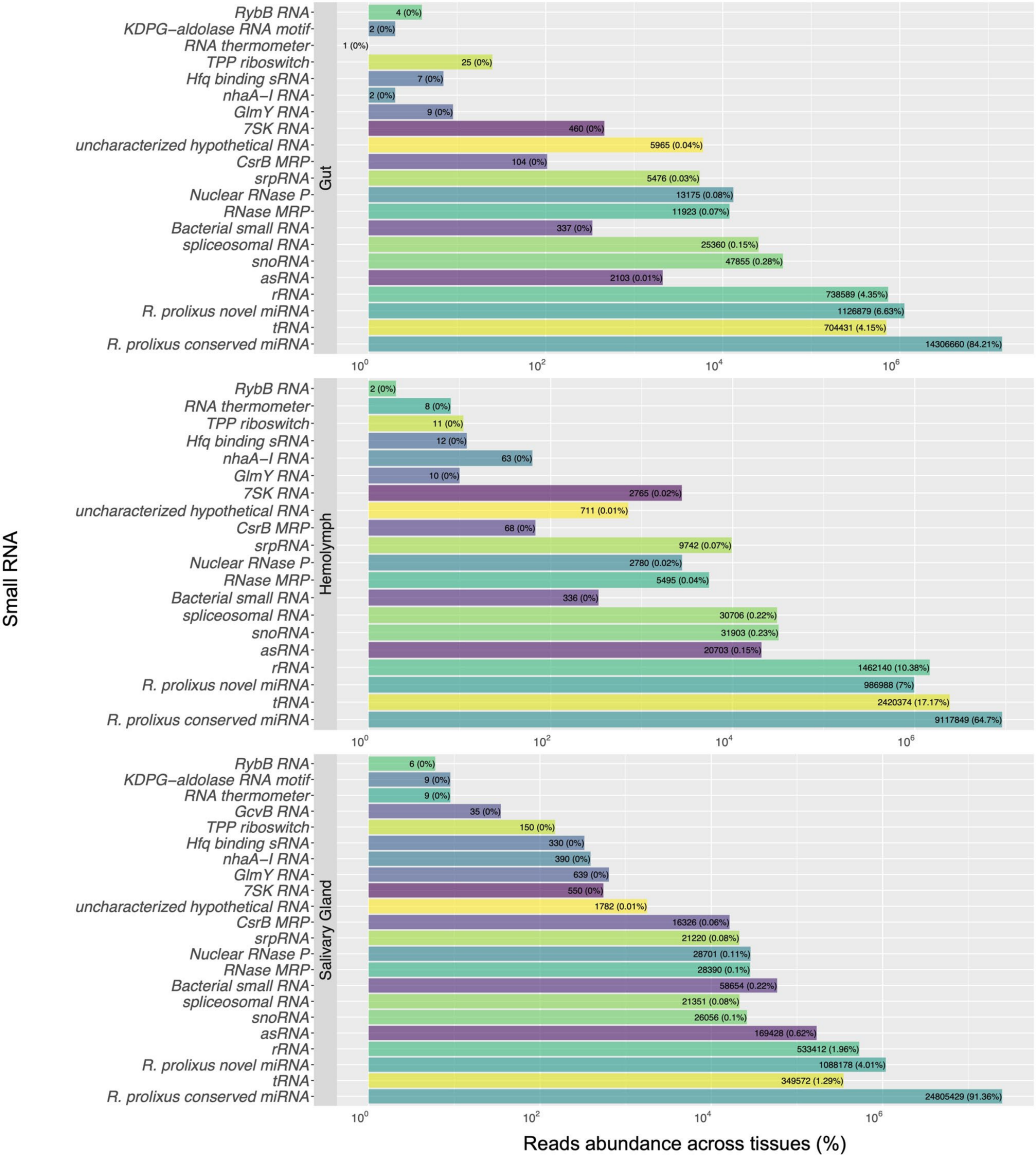
Small RNA abundance of *Rhodnius prolixus* samples. The bar graphs summarize reads abundance of genome-mapped reads aligned against a collection of sncRNAs from Triatominae downloaded from the RNAcentral database (https://rnacentral.org) and the extracted *R. prolixus* miRNAs sequences (conserved and novel). The horizontal axis represents the abundance (%) of aligned reads (log2 of the enrichment ratio).

Furthermore, we performed a second analysis to estimate the abundance of aligned reads, expressed as percentage, which reveals the relative frequency of the identified sncRNA types and that the *R. prolixus* sncRNA collection is enriched with miRNAs. Analysis of read abundance within each sample demonstrated a similar expression pattern in all tissues (Fig. 6). Although miRNAs represented a minor proportion of the sncRNA diversity analysis (Supplementary Fig. S4), conserved miRNAs are the most abundant type of sncRNA in G (84.21%), H (64.7%), and SG (91.36%) tissues (Fig. 6). *R. prolixus* novel miRNAs constitute 6.63% (G), 7% (H), and 4.01% (SG). The remaining fraction of reads corresponds to non-miRNA types, representing a minor proportion of the total sncRNAs.

### In silico prediction of miRNA:mRNA (*Rhodnius prolixus* miRNA:human blood) target site interactions suggests a cross-species gene expression regulation

Triatomines have evolved salivary molecules that specifically target critical immune and hemostatic pathways in the host to enhance the blood-feeding process. Considering that the primary role of miRNAs is to bind to the 3′ UTR of target mRNAs, we employed an in silico approach to assess miRNA:mRNA interactions between vector and host molecules. We focused on the top eight most highly expressed miRNAs in *R. prolixus* SGs, those with AI > 70 (namely miR-283, miR-12a, miR-281, bantam, miR-8, miR-375, miR-276b, and miR-306). mRNA transcripts from human blood were used for target analysis prediction. The overall results revealed that out of 4514 blood mRNA transcripts, 322 were identified as potential targets for the top eight expressed miRNAs from *R. prolixus* SGs, including 72 being targeted by at least two different miRNAs. Notably, multiple targets were observed when a single miRNA targeted the 3′UTRs of different genes. A comprehensive list of the mRNAs targeted by SG miRNAs and the 1128 putative extracted interactions can be observed in Supplementary Table S3. Supplementary Fig. S5 provides the number of interactions.

**Figure 7:**
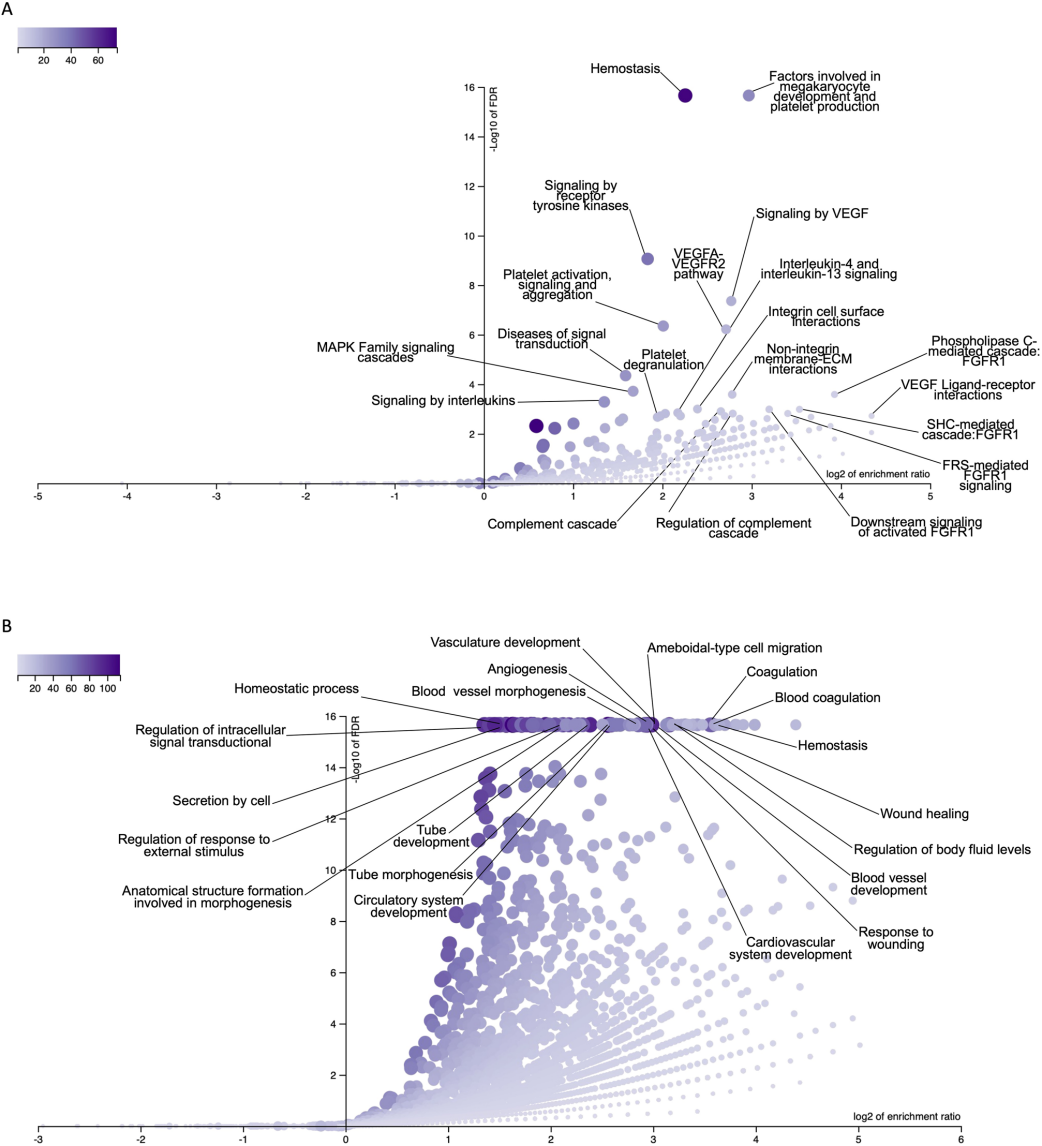
Enriched pathways and Gene ontology Biological Process terms related to genes targeted by the top eight most highly expressed miRNAs from *Rhodnius prolixus* salivary glands. (**A**) Enriched pathways. The horizontal axis represents the Log2 of the enrichment ratio. The vertical axis represents the − Log10 value of the FDR rate. The size and color of the dots are proportional to the size of the category. The most significant pathways are towards the top. Especially meaningful categories for anti-hemostatic salivary function within the TOP 20 functional categories with FDR <  = 0.05 are labeled. (**B**) Enriched set of terms from Geneontology Biological Process. The horizontal axis represents the Log2 of the enrichment ratio. The vertical axis represents the − Log10 value of the FDR rate. The size and color of the dots are proportional to the size of the category. The most significant pathways are towards the top. The TOP 20 terms with FDR <  = 0.05 are labeled.

To gain biological insight from the generated data, we performed an enrichment analysis using Reactome Pathways and Biological Process from Gene Ontology on the set of the target mRNA^80^. The analysis highlighted biological pathways involved in host responses. Meaningful results within the TOP 20 enriched pathways are presented in Fig. 7A. These include Hemostasis, Factors involved in megakaryocyte development and platelet production, Signaling by VEGF, Signaling by receptor tyrosine kinases, Platelet activation, signaling and aggregation, VEGFA-VEGFR2 Pathway, and Complement cascade. Additionally, the enriched set of terms from Gene Ontology related to the targeted genes revealed biological processes involved in hemostasis, coagulation, and vasculature (Fig. 7B). Previous studies on *A. aegypti* showed that five salivary miRNAs (miR-184, miR-375, miR-2490, miR-12 and miR-100) play potential roles in the transmission of Chikungunya virus (CHIKV) and in the establishment of infection during blood feeding on the host^60^. Furthermore, the analysis of salivary miRNAs from *Ixodes ricinus* tick suggests they modulate vertebrate host homeostasis^81^. More recently, a study on the malaria vector *Anopheles coluzzii* revealed the presence of salivary miRNAs that share similarity with human miRNAs, indicating their potential role in regulating host mRNAs associated with immune and inflammatory responses^39^.

These findings support that a dynamic cross-species gene expression regulation mediated by miRNAs from insect vectors may occur within the vector-host and vector-pathogen-host scenarios during blood feeding. Our results indicate that miRNAs from *R. prolixus* saliva can also target host responses, such as the human hemostatic response. Moreover, host exposure to vector bites leads to physiological changes that may be attributed to miRNA's vector molecules, highlighting the potential of circulating miRNAs as biomarkers of vector bites. Incorporating such information provides valuable insight into a new paradigm of salivary molecule functions in hematophagous arthropods. It is important to note that the identification and characterization of individual miRNAs from hematophagous insect vectors and their potential targets have not been fully elucidated to date.

## Conclusion

Here we present the first comprehensive miRNome profiles of *R. prolixus* tissues using high-throughput sequencing. Our findings successfully identified conserved as well as predicted novel miRNAs. The results highlight the ubiquitous presence of conserved miRNAs in *R. prolixus* tissues, with tissue-specific enrichment. Gene expression control is a complex process influenced by multiple regulators. Regarding miRNAs from G and H, they may have a role in blood digestion, metabolism regulation, and immunity. Additionally, our in silico analysis indicates that miRNAs expressed in the SGs could potentially exert a cross-species gene expression regulation when delivered in the vertebrate host at the bite site, specifically targeting the host’s hemostatic responses. It is plausible that these miRNAs may be packaged in exosomes, providing protection against degradation until they reach the host. Although the precise biological functions of *R. prolixus* miRNAs remain a major challenge, their identification provides new insights into the hematophagous vector molecules, offering pieces of knowledge that could be used for the development of novel approaches to the control of Chagas disease and improving our understanding on the intricate vector-parasite-host interactions.

## Materials and methods

### Sample collection and total RNA extraction

*Rhodnius prolixus* triatomines were reared in standard insectary conditions at the University of Brasilia, Brazil (27 ± 1 °C, 70–75% relative humidity, and 12 h/12 h light/dark cycle). The blood source of these insects was *Gallus gallus domesticus*. Adult insects were dissected under a stereomicroscope at 4× magnification in cold sterile phosphate-buffered saline to obtain their SG and G. To collect the H, the end of insect legs was sectioned with scissors, and the fluid was collected using a microtiter pipette. SGs and H samples were collected from 30 individuals at 10-, 20- and 30-days post-blood feeding. The Gs from 20 individuals were collected 15 days post-blood feeding. Tissues were dissected, pooled in separate microtubes containing cold TRIzol® reagent (Invitrogen, Carlsbad, CA, USA), and stored at − 80°°C until total RNA extraction following the manufacturer’s protocol. Following the extraction, samples were treated with turbo-DNase (Ambion, Austin, TX, USA) and quantified using Qubit 2.0 (Invitrogen, USA). RNA integrity was determined by lab-on-chip analysis using the Agilent 2100 Bioanalyzer (Agilent Technologies, Santa Clara, CA, USA) and the three samples were transferred to RNAstable® microtubes (Biomatrica, San Diego, USA).

### Small RNA-seq and bioinformatic analysis

Small RNA extraction, library construction, and sequencing were performed by Macrogen, Inc. (Seoul, South Korea). Individual adapter sequences were added to each tissue pool sample and three cDNA libraries were constructed using the TruSeq Small RNA Library Prep Kit (Illumina Inc., San Diego, CA). Sample libraries were sequenced on an Illumina HiSeq 2500 system (Illumina, San Diego, CA, USA) following a single-end 51  nt strategy. More than 200 million reads were generated across the three libraries. After sequencing, raw reads were checked for data quality using FastQC v0.11.9 (Babraham Institute, Cambridge, UK)^82^. Reads were then trimmed or discarded depending on their quality and size using Cutadapt v3.3^83^. Only processed reads with a minimum of 18 nt and a maximum of 50 nt were maintained. This process yielded high-quality and reliable data. In-house Python scripts were designed to shape the input data along the pipeline as well as R scripts for plots and command lines, which are available at https://github.com/waldeyr/Rhodnius_prolixus_miRNA_project.

The processed reads were mapped to the RprolixusV48 reference genome obtained from the VectorBase (https://vectorbase.org)^84^. For this task, miRDeep2.0.1.2^75^ employed Bowtie v1.2.3^85^ through the *mapper.pl* script. Only perfect alignments were retained (full length, 100% identity). Using the mapped reads and a file (downloaded from miRBase database, release 22.1^56^) containing 8763 known miRNA sequences from Ecdysozoa taxa, *R. prolixus* miRNAs were annotated, and novel miRNAs were predicted using *miRDeep2.pl* script with default options. The miRDeep2 core algorithm evaluated the presence of typical miRNA hairpin structures. If the reads fit in the hairpin as would be expected from Dicer processing, then the potential precursor was assigned as a genuine miRNA. As a constraint to add more reliability to the results, the reads should align to mature (guide strand) and star sequences (defined as the passenger strand base pairing to the potential mature sequence). Complementarily, the quantifier module from the miRDeep2 tool summed up miRNA read counts. The extracted known miRNAs were named according to their most similar miRNAs in the miRBase database. Results were manually curated and integrated into a Microsoft® Excel spreadsheet that contains detailed information about the miRNAs extracted by the miRDeep2 package in the sequencing datasets (Supplementary Table S4). Mature and novel miRNAs were considered high-confidence and selected as valid when filling parameters Significant randfold *p*-value: Yes; miRDeep2 score: >  = 1; Mature read count: > 10; and Star read count: >  = 1.

We assumed the rRNA population is present proportionally to total RNA in tissues and applied a normalization strategy that considers rRNA read counts as a biological internal standard^86,87^. This internal control was used to manage between-sample biases enabling a comparison of miRNA expression levels. The term “abundance index” (AI) was employed to compare the relative expression of miRNAs, which here is defined as the number of reads mapped to total rRNA counts divided by the number of reads mapped to a single miRNA (both known and novel). AI > 70 was set as the enrichment cut-off (miRNAs were considered highly expressed with AI values until tenfold less expressed than miR-283 from SG tissue, AI = 718, the most expressed miRNA across the three tissues). *χ*2 test and *p*-value were calculated to detect the significance level of changes. During the text, the discussed abundance levels are based on this outlined strategy.

In parallel, to classify the sncRNA types in the samples, such as ribosomal RNA (rRNA), Piwi-interacting RNA (piRNA), transfer RNA (tRNA), small nuclear RNA (snRNA), and small nucleolar RNA (snoRNA); the reads that mapped to the *R. prolixus* genome were also aligned against Triatominae non-coding RNAs downloaded from the RNAcentral database release 10.0^88^ using Blast^89^. The graphical representations of miRNAs secondary structures were obtained by using RNAfold with default settings^90^. The two most abundant miRNAs of each tissue were selected for this analysis. The best structure for each sequence was selected based on minimum free energy values.

### In-silico mapping of *Rhodnius prolixus* miRNAs to human blood transcripts

miRNA target prediction was conducted using the miRNAconsTarget tool from sRNAtoolbox (https://arn.ugr.es/srnatoolbox)^91^, which employs four miRNA target prediction programs: TargetSpy, miRanda, PITA, and Seed2. Only miRNA:mRNA interactions predicted by the four programs were considered. A target is only considered a consensus if all four methods predict an overlapping interaction in the same region inside the targeted sequence. The prediction was done using the eight most abundant miRNAs expressed in the SGs and a set of 3′UTRs (≥ 30 nt) of mRNAs from human blood downloaded from Human Protein Atlas as input files^92^. mRNA blood transcripts were downloaded from Ensembl, using the BioMart tool^93^. An over-representation analysis (ORA) method available in the WebGestalt tool^94^ was used to perform enrichment analysis of the set of the predicted targeted genes to gain insight into enriched metabolic pathways in Reactome^95^ and biological processes in Gene Ontology^80,96^. The parameters used were as follows: option “Top 20” on “Significance Level”, meaning the categories are first ranked based on the FDR threshold, and then the 20 most significant categories are selected; 0.05 on FDR threshold; and BH on Multiple Test Adjustment.

### Small RNA extraction and RNASeq results validation by RT-qPCR amplification

To establish a correlation between RT-qPCR Ct values and read abundance from small RNA-seq, it was necessary to choose identified miRNAs that covered a range of expression levels, including both high and low abundance^39^. Five highly-abundant salivary miRNAs (miR-283, miR-12a, miR-281, bantam, miR-8; AI ≥ 500) and two lowly-abundant miRNAs (miR-315, miR-92b; AI < 7) were selected. The validation of this subset was performed using the Stem-loop Reverse-Transcription Polymerase Chain Reaction (slRT-PCR) strategy^74^. For this purpose, 90 SGs pairs were collected in 500 µL of TRIzol™ Reagent (Invitrogen®) as described above, in Sample Collection Methods. The small RNAs (< 50 nt) were isolated from the tissue using RNeasy Mini Kit (QIAGEN®) according to the manufacturer’s guidelines. First-strand cDNA synthesis was generated in a 20 μL reaction volume from 4 µL of small RNA using the GoScript™ Reverse Transcription System (Promega®) according to the manufacturer's instructions and the specific stem-loop primers (1 μM).

Reactions of RT-qPCR amplification were performed in a final volume of 12 μL, comprising 2× GoTaq® qPCR Master Mix (Promega®), 0.12 µL of Supplemental CXR Reference Dye, the specific forward and universal reverse primers (0.5 μM each) and 0.25 μL of the specific first strand cDNA reactions. The reactions were conducted using the StepOnePlus Real-Time PCR System (Applied Biosystem®). Amplification was performed as follows: polymerase activation stage of 2 min at 95 °C followed by 45 cycles (15 s at 95 °C, 1 min at 60 °C). Melting curves were obtained for each miRNA to verify the absence of unspecific amplification products with detection steps every 0.8% temperature increment. All reactions were performed in triplicate. The degree of linear association between read counts and Ct values was measured by calculating the Pearson coefficient correlation using GraphPad Software®, San Diego California USA. To estimate the strength of the relationship between data, p-value (derived from t-test) was used. A list of primer sequences, including the specific stem-loop and reverse primers, as well as the universal primer can be accessed in Supplementary Table S5.

### Supplementary Information

Supplementary Figure S1.

Supplementary Figure S2.

Supplementary Figure S3.

Supplementary Figure S4.

Supplementary Figure S5.

Supplementary Table S1.

Supplementary Table S2.

Supplementary Table S3.

Supplementary Table S4.

Supplementary Table S5.

## Data Availability

Small RNA-seq raw datasets were deposited in the National Center for Biotechnology Information (NCBI) in the Sequence Read Archive (SRA) under BioProject ID: PRJNA925536, Biosamples SAMN32800154 (G), SAMN32800155 (H) and SAMN32800156 (SG); and Sequence Read Archive (SRA) accessions SRR23130199, SRR23130198, and SRR23130197.
