# Supplementary figures and images for: Insights into the microRNA landscape of Rhodnius prolixus, a vector of Chagas disease
Source: Sci Rep. 2023 Aug 12;13:13120. doi: 10.1038/s41598-023-40353-9 (PMC10423254; doi:10.1038/s41598-023-40353-9)

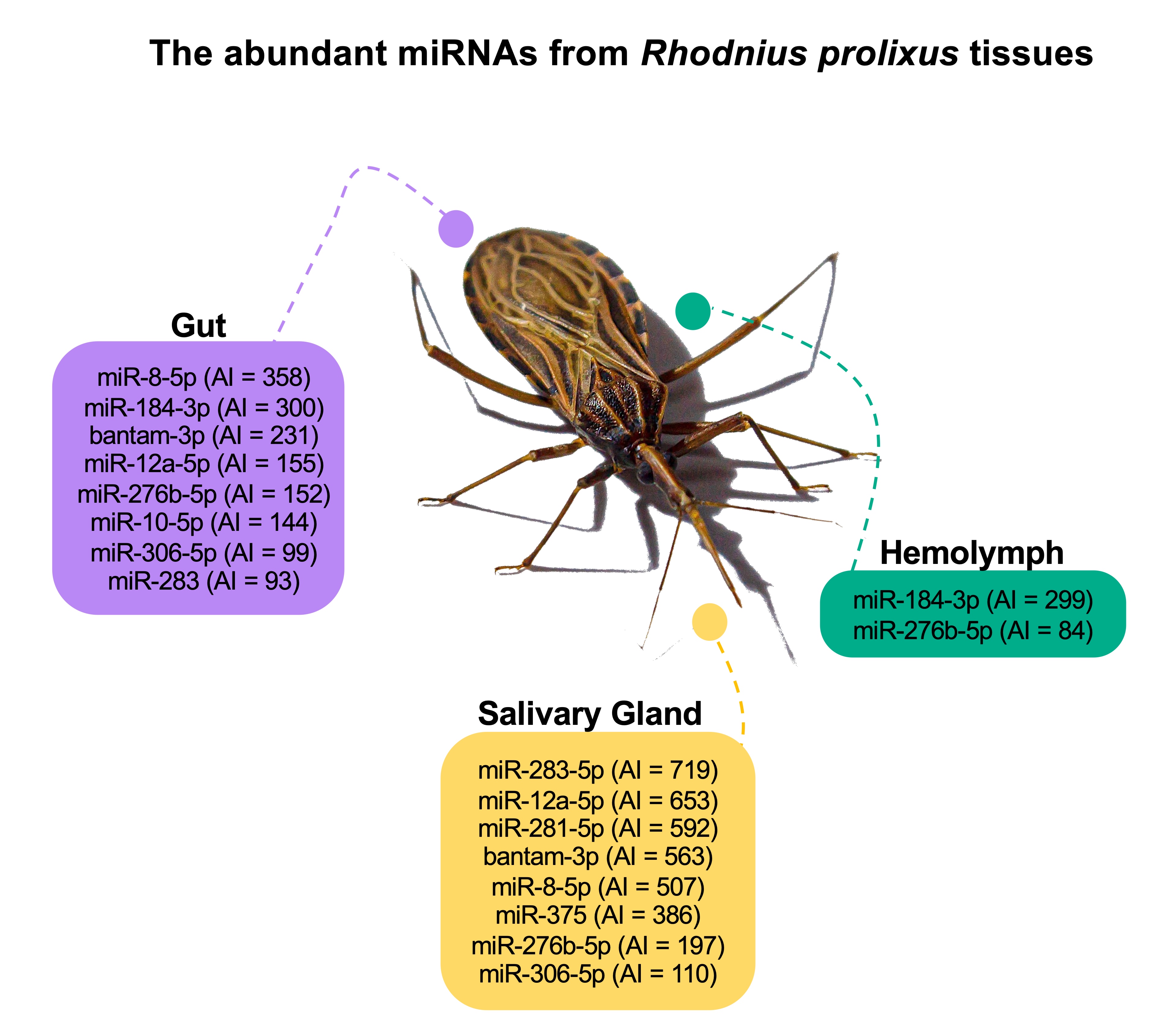

Supplement: Supplementary file 1 — Supplementary Figure S1. [file 41598_2023_40353_MOESM1_ESM.jpg]

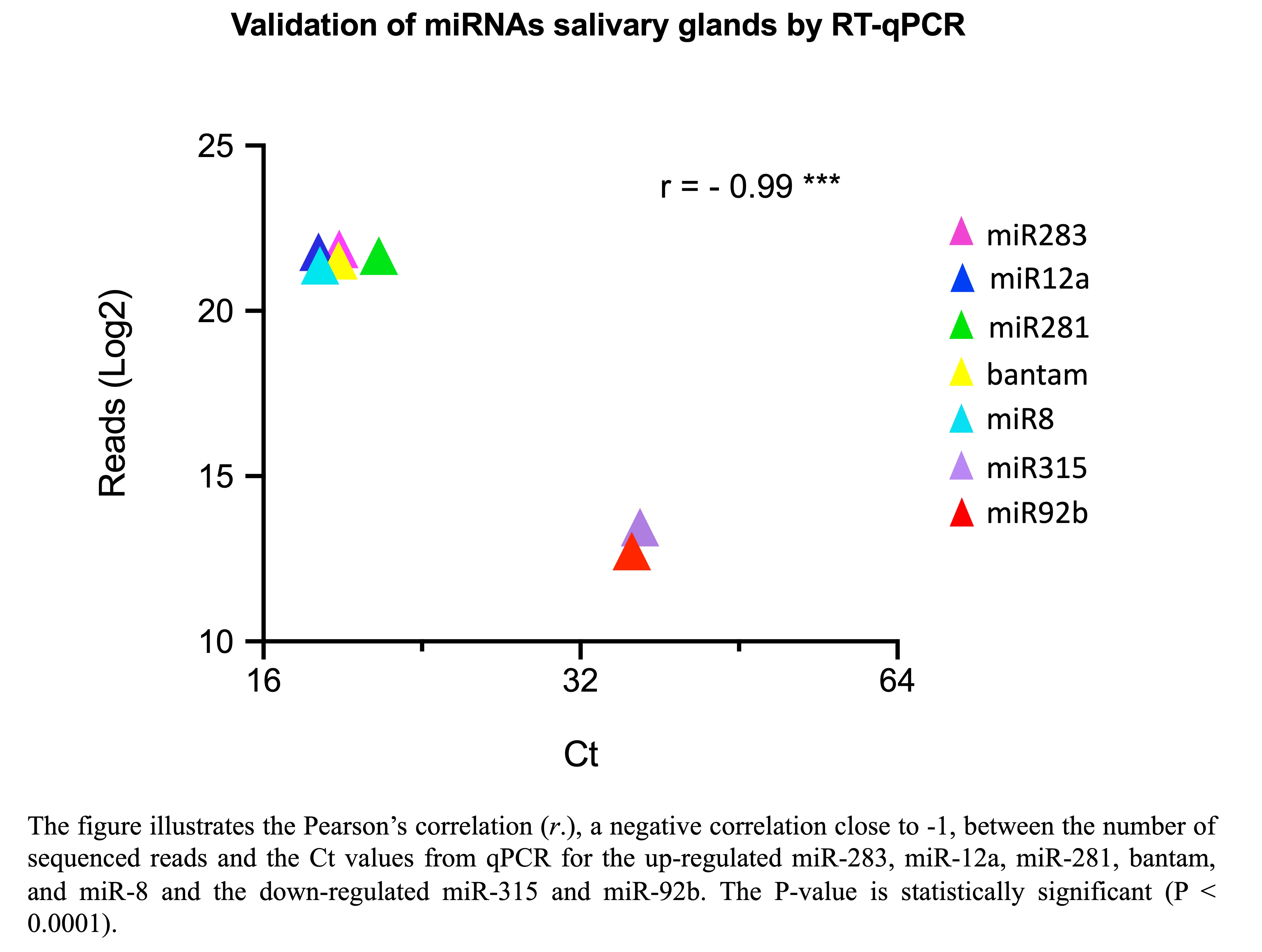

Supplement: Supplementary file 2 — Supplementary Figure S2. [file 41598_2023_40353_MOESM2_ESM.jpg]

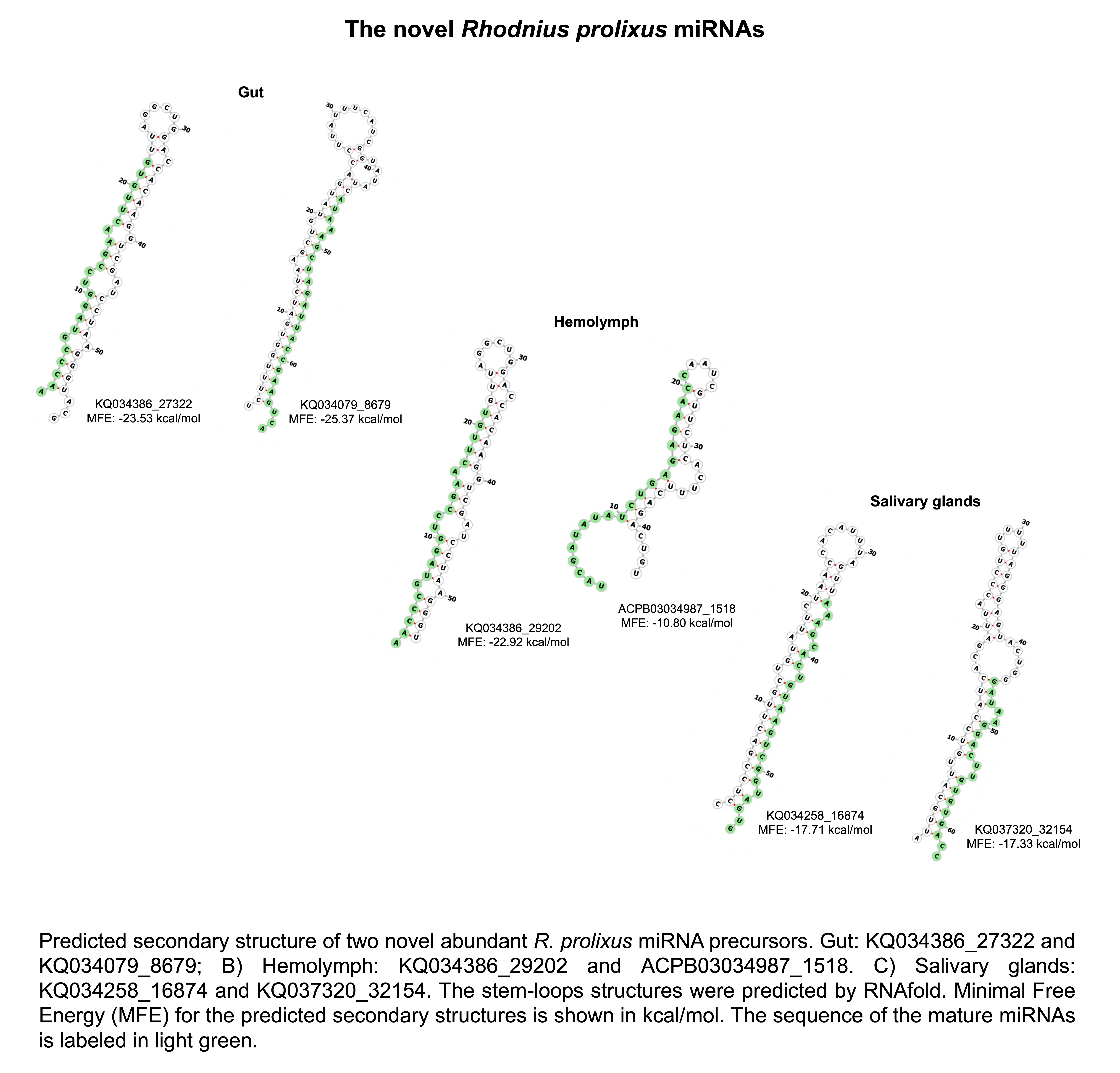

Supplement: Supplementary file 3 — Supplementary Figure S3. [file 41598_2023_40353_MOESM3_ESM.jpg]

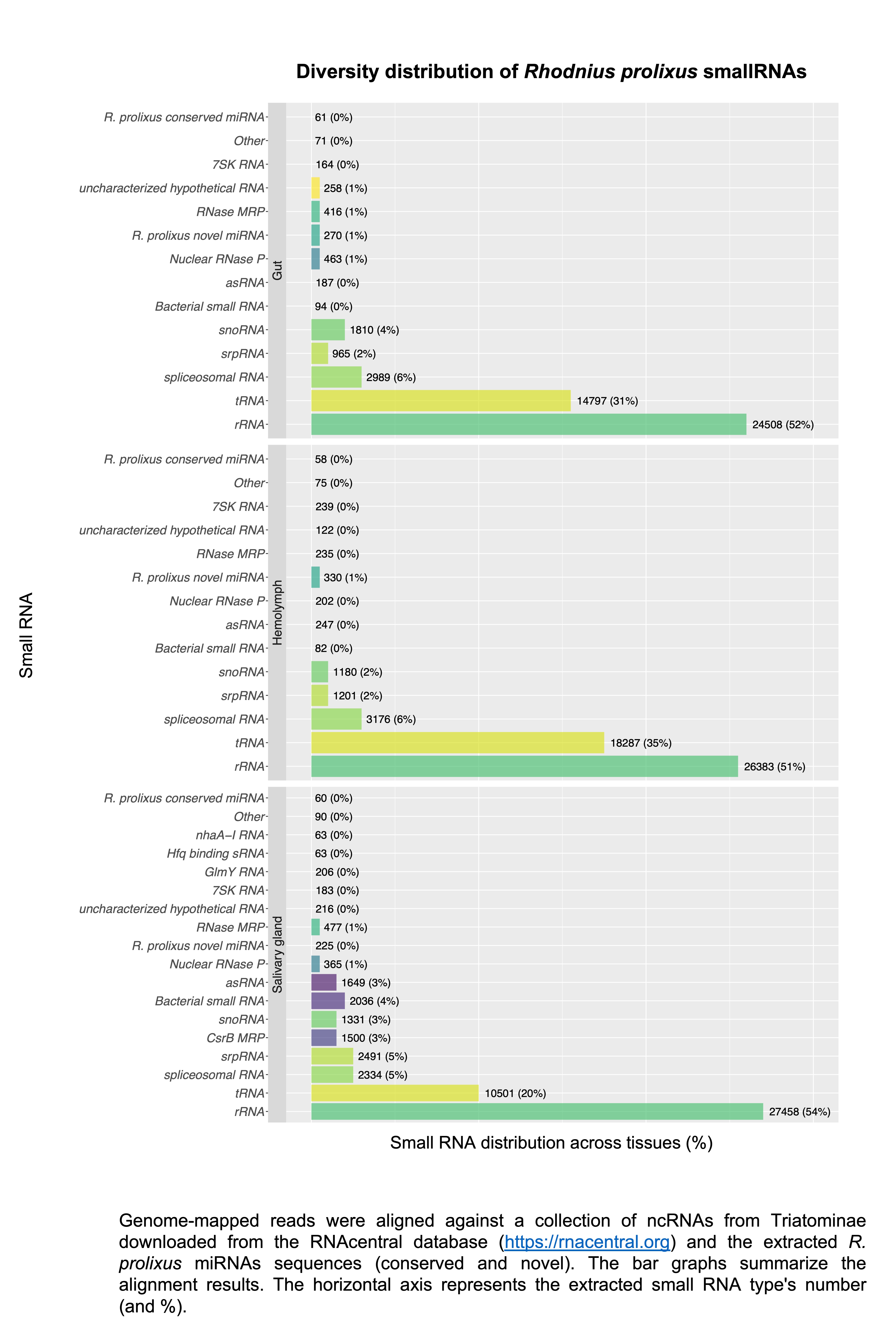

Supplement: Supplementary file 4 — Supplementary Figure S4. [file 41598_2023_40353_MOESM4_ESM.jpg]

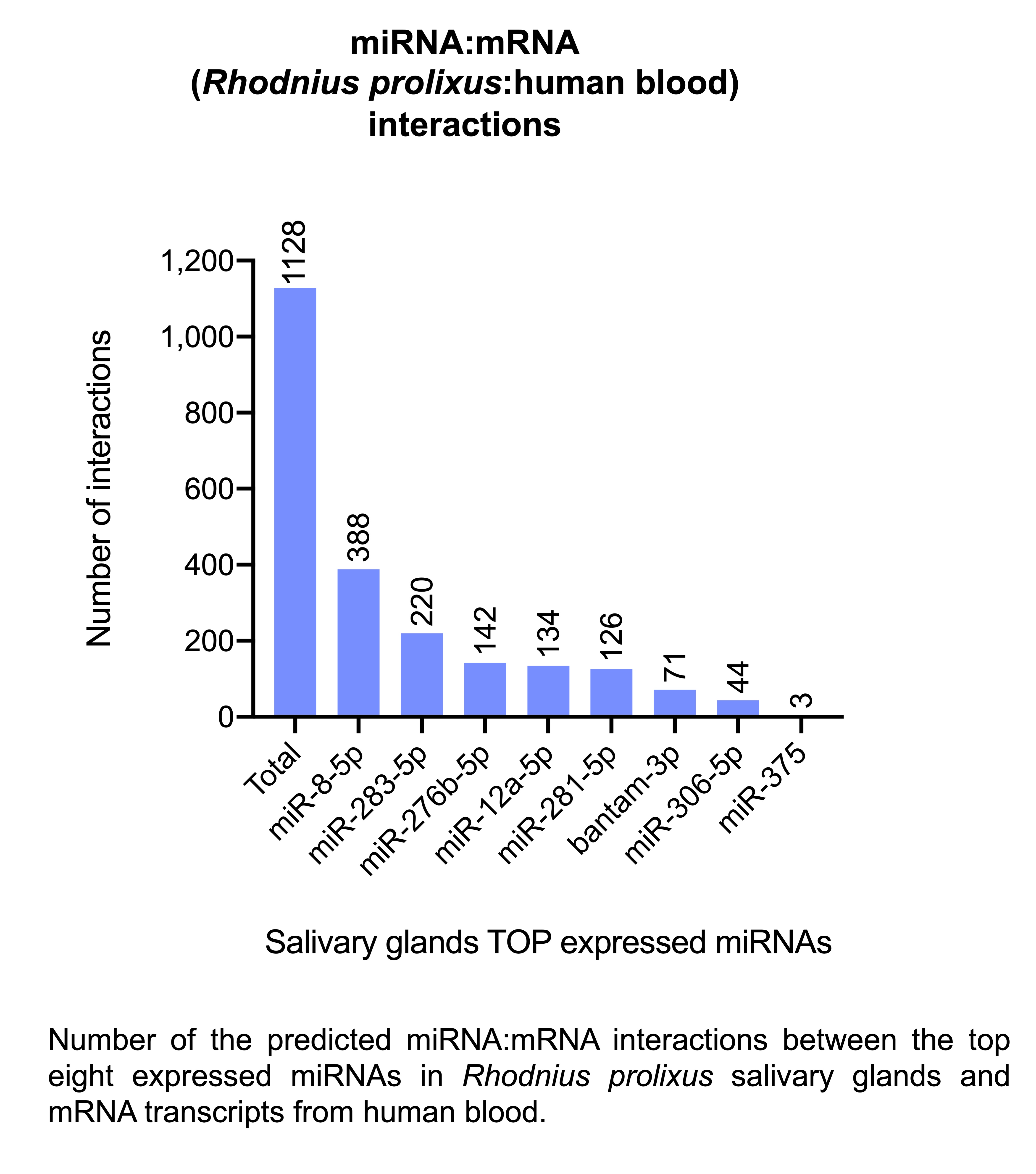

Supplement: Supplementary file 5 — Supplementary Figure S5. [file 41598_2023_40353_MOESM5_ESM.jpg]
