# Supplementary material for: Insights into the microRNA landscape of Rhodnius prolixus, a vector of Chagas disease
Source: Sci Rep. 2023 Aug 12;13:13120. doi: 10.1038/s41598-023-40353-9 (PMC10423254; doi:10.1038/s41598-023-40353-9)
Supplement: Supplementary file 6 — Supplementary Table S1. [file 41598_2023_40353_MOESM6_ESM.docx]

**Supplementary Table S1**  **Small RNA sequencing results from *Rhodnius prolixus* samples**

| Sample | Raw reads^1^ | Processed reads^2^ | Q20 (%)^3^ | Mapped reads^4^ | Mapped miRNA reads^5^ |
| --- | --- | --- | --- | --- | --- |
| Gut | 63,286,547 | 61,927,826  (97.85%) | 98.48 | 1,020,324 | 21,509 |
| Hemolymph | 57,623,029 | 38,714,067  (67.19%) | 98.29 | 1,032,644 | 34,392 |
| Salivary glands | 80,042,912 | 54,881,237  (68.56%) | 98.43 | 1,068,947 | 22,096 |

**^1^**Total raw read count.

**^2^**Processed read count. Total reads remaining after adapter removal and size selection (≥18 bp).

**^3^**Ratio of bases with Phred quality score equal to or greater than 20 of processed reads.

**^4^**Processed reads that mapped to the RprolixusV48 reference genome (obtained in <https://vectorbase.org>).

**^5^**Processed unique reads annotated as miRNA that mapped to the RprolixusV48 reference genome (obtained in https://vectorbase.org)
