# Supplementary material for: Insights into the microRNA landscape of Rhodnius prolixus, a vector of Chagas disease
Source: Sci Rep. 2023 Aug 12;13:13120. doi: 10.1038/s41598-023-40353-9 (PMC10423254; doi:10.1038/s41598-023-40353-9)
Supplement: Supplementary file 7 — Supplementary Table S2. [file 41598_2023_40353_MOESM7_ESM.docx]

**Supplementary Table S2 Reads from *Rhodnius prolixus* samples identified as novel miRNAs by miRDeep2**

| Sample | Total read count | Mature read count | Loop read count^1^ | Star read count^2^ | Total Novel miRNAs^3^ |
| --- | --- | --- | --- | --- | --- |
| Gut | 675,596 | 613,476 | 57 | 62,063 | 112 |
| Hemolymph | 764,323 | 746,750 | 1,453 | 16,120 | 73 |
| Salivary glands | 661,471 | 627,405 | 227 | 33,839 | 78 |

**^1^**Loop is defined as the sequence between the mature and star sequence

**^2^**Star sequence is defined as the sequence base pairing to the mature sequence

**^3^**Number of novel miRNAs detected by miRDepp2 against miRbase mature reference database Ecdysozoa within the following parameters: number of mature read count > 10; number of star read count equal to or greater than 1; miRDepp2 score equal to or exceeding the cut-off of 1; “yes” in significant randfold p-value.
