# Supplementary material for: Insights into the microRNA landscape of Rhodnius prolixus, a vector of Chagas disease
Source: Sci Rep. 2023 Aug 12;13:13120. doi: 10.1038/s41598-023-40353-9 (PMC10423254; doi:10.1038/s41598-023-40353-9)
Supplement: Supplementary file 10 — Supplementary Table S5. [file 41598_2023_40353_MOESM10_ESM.docx]

**Supplementary Table S5: List of primers used for reverse transcription and qPCR**

| **miRNA** | **Primer** | **Sequence** |
| --- | --- | --- |
| miR-283 | Stem-Loop RT primer | 5′ GTC GTA TCC AGT GCA GGG TCC GAG GTA TTC GCA CTG GAT ACG AC TCA GAA 3′ |
|  | Forward primer | 5' TGG GCG CAA ATA TCA GCT GGT AAT 3' |
| miR-12a | Stem-Loop RT primer | 5′ GTC GTA TCC AGT GCA GGG TCC GAG GTA TTC GCA CTG GAT ACG AC ATC AGT 3′ |
|  | Forward primer | 5' TGG GCG GTG AGT ATT ACA TCA GGT 3' |
| miR-281 | Stem-Loop RT primer | 5′ GTC GTA TCC AGT GCA GGG TCC GAG GTA TTC GCA CTG GAT ACG AC ACT GTC 3′ |
|  | Forward primer | 5' CAC GCA AAG AGA GCT ATC CGT CG 3' |
| [bantam](http://www.mirbase.org/cgi-bin/query.pl?terms=pca-bantam-3p\|MIMAT0045827\|Polistes) | Stem-Loop RT primer | 5′ GTC GTA TCC AGT GCA GGG TCC GAG GTA TTC GCA CTG GAT ACG AC ATC AGC 3′ |
|  | Forward primer | 5' TGG GCG CTG AGA TCA TTG TGA AAG 3' |
| miR-8 | Stem-Loop RT primer | 5′ GTC GTA TCC AGT GCA GGG TCC GAG GTA TTC GCA CTG GAT ACG AC CAT CAT 3′ |
|  | Forward primer | 5' GGC GCG CTA ATA CTG TCA GGT AAT 3' |
| miR-315 | Stem-Loop RT primer | 5′ GTC GTA TCC AGT GCA GGG TCC GAG GTA TTC GCA CTG GAT ACG AC AGG CTT 3′ |
|  | Forward primer | 5' TCG GCG CTT TTG ATT GTT GCT CAG 3' |
| miR-92b | Stem-Loop RT primer | 5′ GTC GTA TCC AGT GCA GGG TCC GAG GTA TTC GCA CTG GAT ACG AC CAG GCC 3′ |
|  | Forward primer | 5' CCA GGA AAT TGC ACT AGT CCC GG 3' |
| Universal Reverse | Uni_Rev | 5’ CCA GTG CAG GGT CCG AGG TA 3’ |
